# Integrating ^68^Ga-PSMA-11 PET/CT with Clinical Risk Factors for Enhanced Prostate Cancer Progression Prediction

**DOI:** 10.3390/cancers17142285

**Published:** 2025-07-09

**Authors:** Joanna M. Wybranska, Lorenz Pieper, Christian Wybranski, Philipp Genseke, Jan Wuestemann, Julian Varghese, Michael C. Kreissl, Jakub Mitura

**Affiliations:** 1 Division of Nuclear Medicine, Department of Radiology & Nuclear Medicine, Faculty of Medicine, Otto von Guericke University Magdeburg, 39120 Magdeburg, Germanyjakub.mitura@med.ovgu.de (J.M.); 2 Institut für Medical Data Science, Medical Faculty, Otto von Guericke University Magdeburg, 39120 Magdeburg, Germany

**Keywords:** prostate cancer, ^68^Ga-PSMA-11 PET/CT, SUVmax, CAPRA score, early biochemical recurrence, outcome prediction

## Abstract

Background/Objectives: This study evaluates whether combining ^68^Ga-PSMA-11-PET/CT derived imaging biomarkers with clinical risk factors improves the prediction of early biochemical recurrence (eBCR) or clinical progress in patients with high-risk prostate cancer (PCa) after primary treatment, using machine learning (ML) models. Methods: We analyzed data from 93 high-risk PCa patients who underwent ^68^Ga-PSMA-11 PET/CT and received primary treatment at a single center. Two predictive models were developed: a logistic regression (LR) model and an ML derived probabilistic graphical model (PGM) based on a naïve Bayes framework. Both models were compared against each other and against the CAPRA risk score. The models’ input variables were selected based on statistical analysis and domain expertise including a literature review and expert input. A decision tree was derived from the PGM to translate its probabilistic reasoning into a transparent classifier. Results: The five key input variables were as follows: binarized CAPRA score, maximal intraprostatic PSMA uptake intensity (SUVmax), presence of bone metastases, nodal involvement at common iliac bifurcation, and seminal vesicle infiltration. The PGM achieved superior predictive performance with a balanced accuracy of 0.73, sensitivity of 0.60, and specificity of 0.86, substantially outperforming both the LR (balanced accuracy: 0.50, sensitivity: 0.00, specificity: 1.00) and CAPRA (balanced accuracy: 0.59, sensitivity: 0.20, specificity: 0.99). The decision tree provided an explainable classifier with CAPRA as a primary branch node, followed by SUVmax and specific PET-detected tumor sites. Conclusions: Integrating ^68^Ga-PSMA-11 imaging biomarkers with clinical parameters, such as CAPRA, significantly improves models to predict progression in patients with high-risk PCa undergoing primary treatment. The PGM offers superior balanced accuracy and enables risk stratification that may guide personalized treatment decisions.

## Simple Summary

In this study, we explored the use of machine learning (ML) to improve the prediction of early prostate cancer (PCa) progression by combining ^68^Ga-PSMA-11 PET/CT imaging biomarkers with clinical risk factors from 93 high-risk PCa patients. The CAPRA score served as a comparator. We developed a probabilistic graphical model (PGM) and a logistic regression (LR) model. Key predictors used in both models included the CAPRA-based score and SUVmax, followed by bone metastases, seminal vesicle infiltration, and nodal involvement at common iliac bifurcation. The PGM outperformed both the CAPRA score and the LR model, achieving a balanced accuracy of 0.73. Our findings demonstrate that an ML-derived PGM integrating ^68^Ga-PSMA-11 PET/CT imaging with clinical data significantly enhances early PCa progression prediction.

## 1. Introduction

Prostate cancer (PCa) is a major global health problem, ranking as the second most frequently diagnosed malignancy and the fifth leading cause of cancer-related mortality among men worldwide [1,2]. Accurate risk stratification is crucial to aligning medical care with disease aggressiveness. Current guidelines from the European Association of Urology (EAU), the National Comprehensive Cancer Network (NCCN), and the American Urological Association (AUA) underscore the need for reliable prognostic tools to guide personalized care, minimize overtreatment in low-risk cases, and intensify surveillance and management for patients with PCa with advanced tumors or high-risk features for recurrence [3,4,5,6].

Several predictive models have been validated to support these clinical decisions. Among the most widely adopted are the Surgical Cancer of the Prostate Risk Assessment (CAPRA-S) or the Japan Cancer of the Prostate Risk Assessment (J-CAPRA) scores, which estimate the risk of early biochemical recurrence (eBCR), early clinical progression, metastatic spread, and cancer-related mortality in patients after radical prostatectomy or hormonal therapy using clinical and pathological variables [7,8,9]. However, these tools refer to uniform treatment groups and primarily provide discrete risk probabilities at fixed time intervals, such as 5 or 10 years, which may not align with the short- and intermediate-term decision points that clinicians often face in daily practice. In addition, these models are constrained by the availability and quality of clinical data that can limit their utility. A major disadvantage of these models is that they do not include molecular imaging findings, which are becoming increasingly important in the staging and prognosis of PCa.

Prostate-specific membrane antigen positron emission tomography/computed tomography (^68^Ga-PSMA-11  PET/CT) has emerged as a highly sensitive imaging modality that not only outperforms conventional imaging such as CT in the detection of local, nodal, and distant PCa lesions [10,11,12] but also allows quantitative analysis. Therefore, hybrid models that combine clinical risk scores with PSMA-based imaging biomarkers are increasingly being advocated to achieve individualized PET/CT-guided risk estimates [13,14]. However, the prognostic value of the molecular status of the primary tumor, even when combined with CAPRA-S and PSMA-confirmed extraprostatic disease, remains uncertain, as studies have shown inconsistent associations with results such as eBCR-free survival [15].

Although logistic regression (LR) has traditionally been used for modeling tabular clinical data due to its simplicity and interpretability [14], newer approaches increasingly rely on more flexible models such as gradient-boosted machines (GBM) and probabilistic graphical models (PGM). These models can better handle non-linearities and missing data, common traits present in real-world clinical datasets [16]. However, given persistent concerns about reproducibility and variable performance, it remains inconclusive whether machine learning (ML) approaches consistently provide added value over LR models and classical scores such as CAPRA and its equivalents [17].

We hypothesize that integrating PSMA-derived features—such as the intraprostatic maximum standardized uptake value (SUVmax)—alongside established clinical risk factors into an LR model and an ML-derived PGM improves the prediction of early PCa progression in patients after primary treatment. Our objective, therefore, was to compare the performance of the LR model and the PGM using this integrated approach and to test both models against conventional CAPRA-based scoring. Sensitivity, specificity, and balanced accuracy were used as key validation metrics.

## 2. Materials and Methods

### 2.1. Participants and Study Design

This single-center retrospective study included data from 93 patients with histopathologically confirmed high-risk prostate cancer (PCa) who received primary treatment based on clinical evaluation, measurement of prostate-specific antigen (PSA), and imaging results. According to the German S3 PCa Guideline (version 6.2), high-risk PCa is defined by at least one of the following criteria: PSA level > 20 ng/mL, Gleason score ≥ 8, or clinical stage ≥ T2c.

Tissue diagnosis was obtained prior to ^68^Ga-PSMA-11 PET/CT imaging and treatment by systematic or targeted prostate biopsy. Pathological samples were evaluated by institutional uropathologists according to the grading system of the International Society of Urological Pathology (ISUP, 2014) [18]. The cases were categorized according to the Gleason score, with a clinically significant PCa defined as ISUP Grade Group ≥ 2.

The exclusion criteria were as follows: (i) prior local or systemic PCa treatment, (ii) age under 18 years, (iii) presence of another concurrent malignancy at the time of treatment, and (iv) an interval of less than 30 days between PET/CT imaging and documented clinical outcome.

**Figure 1:**
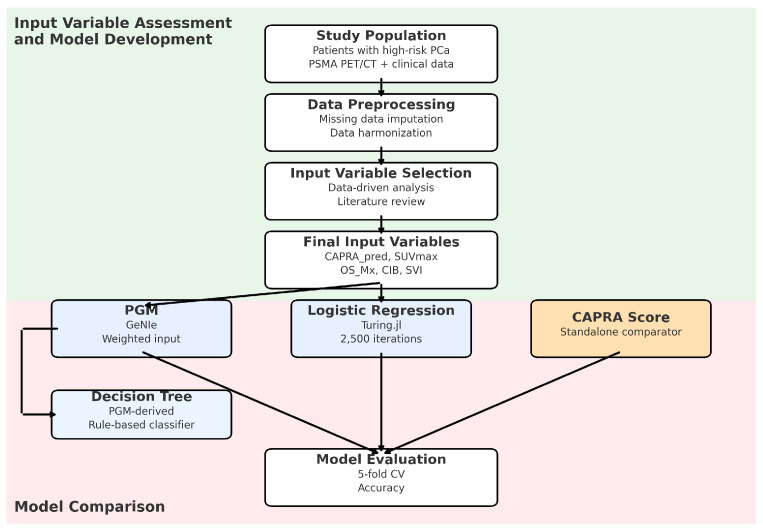
Study flow chart illustrating the development of two predictive models (LR and PGM) using harmonized clinical and ^68^Ga-PSMA-11 PET/CT data (same input variables), alongside a CAPRA-based comparator. Input variable selection integrated data-driven methods and domain knowledge. All models were evaluated using five-fold cross-validation. Finally, a decision tree was derived from the PGM to improve interpretability.

Anonymized datasets were prepared according to the Transparent Reporting of a Multivariate Predictive Model for Individual Prognosis Or Diagnosis (TRIPOD) guidelines [19]. A comprehensive set of clinical and imaging variables (potential input variables) was collected, from which a subset of key predictors was selected to train the LR model and the PGM. The CAPRA score was chosen as an additional standalone comparator of the predictive models. All key methodological steps are summarized in the study flow chart (Figure 1).

The institutional ethics committee approved this study on 21 May 2024 (approval number: 79/24 RAD399). Written informed consent was obtained from all participants.

### 2.2. Imaging Protocol and Evaluation

^68^Ga-PSMA-11 PET/CT images were acquired with a Siemens Biograph mCT 128 PET/CT scanner (Siemens Healthineers, Erlangen, Germany) between 1 January 2022, and 31 December 2023, at the Division of Nuclear Medicine in the Medical University of Magdeburg, Germany.

The [68Ga]Ga-PSMA-11 tracer used in this study was produced on site in the nuclear medicine radiopharmacy laboratory using a generator-based production system with PSMA-11 kits. Image acquisition was performed according to the current guidelines of the European Association of Nuclear Medicine (EANM). A total of 2 MBq/kg body weight of [^68^Ga]Ga-PSMA-11 was administered intravenously (IV). Following a 90–120 min uptake phase, a whole-body PET scan was acquired, extending from the skull base to the upper third of the mid-thigh. A low-dose CT scan was performed for attenuation correction and anatomical localization, after IV administration of 20 mL of iodinated contrast agent for ureteral visualization in most cases. PET/CT data were used not only for primary staging but also to infer anatomical correlates in cases of incomplete clinical or pathological information.

**Figure 2:**
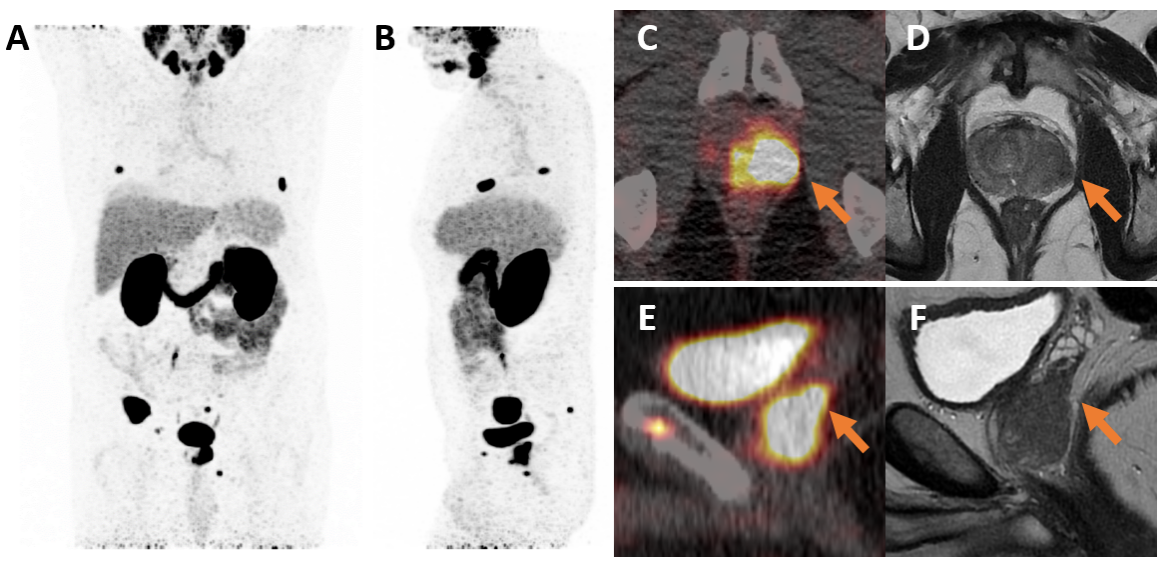
^68^Ga-PSMA-11 PET/CT staging and multiparametric MRI of the prostate in a 68-year-old patient with high-risk prostate cancer (Gleason score 8, initial PSA 20.3 ng/mL) prior to therapy planning. (**A**,**B**) Maximum intensity projection (MIP) PET images (frontal and lateral) demonstrating multiple bone metastases and a clearly visualized locally advanced primary prostate tumor. (**C**) Axial PET/CT demonstrating PSMA ligand uptake in the intraprostatic tumor. (**D**) Axial T2-weighted MRI showing a hypointense tumor in the left prostate lobe with evidence of extraprostatic extension (arrows). (**E**) Sagittal PET/CT reconstruction showing PSMA ligand uptake in the intraprostatic tumor and left seminal vesicle. (**F**) Sagittal T2-weighted MRI illustrating infiltration of the left seminal vesicle (arrows).

The PET/CT images were read and analyzed independently by two board-certified nuclear medicine physicians with more than 3 and 15 years of clinical experience, respectively. Any discrepancies were finally resolved by consensus reading. Figure 2 shows an exemplary ^68^Ga-PSMA-11 PET/CT staging and multiparametric magnetic resonance imaging findings in a patient with high-risk prostate cancer with locally advanced intraprostatic tumor and multiple bone metastases.

### 2.3. Patient Characteristics and Follow-Up

Follow-up data for early clinical progress of PCa and eBCR were collected over a median period of 302 days (range: 30–777 days) after initial staging with ^68^Ga-PSMA-11 PET/CT, in line with the current consensus of the European PCa Guidelines Panel-2024 Update [3]. The demographic and clinical data of the patient, along with the molecular tumor characteristics, are summarized in Table 1. Further [^68^Ga]Ga-PSMA-11 imaging findings and treatment outcomes are shown in Table 2 and Table 3.

### 2.4. Input Variable Assessment

Potential input variables for the development of the predictive model were recorded at the initial diagnosis and PET/CT staging. They are summarized in Table 4. The findings of [^68^Ga]Ga-PSMA-11 were used as a surrogate for missing clinical information, such as SVI, for the CAPRA-S and J-CAPRA risk scores. The final CAPRA scores were then constructed by adding standardized point values for their respective clinical parameters (capra_pred).

The relationship of potential input variables with early PCa progression was statistically assessed using logarithmic regression. The absolute magnitude of the standardized coefficients was used as a surrogate to rank the variables based on their relative importance. To evaluate redundancy, Pearson’s pairwise correlations were calculated. In addition, Spearman rank correlation was applied to identify monotonic associations between input variables and PCa progression. A *p*-value < 0.05 was considered statistically significant for all tests.   

### 2.5. Input Variable Selection

A total of five input variables were chosen for the development of the predictive models, aiming to balance the predictive power and model simplicity and to mitigate the risk of overfitting, which might degrade the model performance in our small and unbalanced dataset [20,21].

Purely data-driven approaches, while effective in pattern detection, often do not reflect the underlying relationships in small or unbalanced clinical datasets [22]. To ensure clinical relevance and reduce overfitting, we adopted a hybrid feature selection strategy that combined statistical analysis, review of the literature, and expert input [22,23,24]. This approach represents a form of manual Knowledge-Driven Feature Engineering (mKDFE), deliberately integrating domain expertise alongside data-driven methods [25].

The final input variables were binarized using predefined thresholds grounded in the literature (see the detailed description in Appendix A).

### 2.6. Outcome Definition

Our models were developed to discriminate patients in the high-risk group with progressive disease (PD) from patients with complete response (CR), partial response (PR), or stable disease (SD) based on a classification of four categories of clinical response. To diagnose PD, early PCa progression had to be confirmed by eBCR, imaging results, or both.

### 2.7. Model Development

We developed two predictive models to estimate the risk of early progression of PCa in high-risk patients: (i) an LR model and (ii) an ML-derived PGM. Both models used the same set of key input variables.

The LR model was chosen to provide a comparative benchmark for the performance of our PGM due to its well-established calibration properties and strong theoretical foundation, particularly when dealing with moderate-sized datasets [26]. It was implemented using the Julia programming language (Version 1.11) and the Turing.jl probabilistic programming package, employing sequential Monte Carlo sampling with 2500 iterations.

The PGM was developed using GeNIe Academic Version 5.0.53.10.0 (BayesFusion, Pittsburgh, PA, USA). The GeNIe software enabled manual assignment of numeric influence weights, which were iteratively refined based on the literature evidence and expert input to optimize the predictive performance across validation folds (see the detailed description in Appendix A). The PGM was trained with expectation maximization and a confidence level of 20 for manually set prior distributions.

### 2.8. Model Comparison

Both models—LR model and PGM—were compared to each other and to the conventional CAPRA score as a standalone predictor to validate the models’ performance. It is widely adopted and well-validated in clinical routine; however, it does not include molecular imaging findings [7,8]. We calculated the CAPRA score (CAPRA-S or J-CAPRA) for each case and binarized the results (capra_pred) taking into account a probability threshold of 0.16 for the progression of PCa according to [8,9] (see the detailed description in Appendix A).

Given the moderate sample size (*n* = 93), we used five-fold cross-validation to evaluate the model performance. Both models—the LR model and the PGM—and the comparator CAPRA score were assessed using standard binary classification metrics, including sensitivity, specificity, and balanced accuracy. This approach enabled a direct comparison between the LR model, the PGM, and the CAPRA score.

### 2.9. Decision Tree

To enhance the interpretability and potential clinical applicability, a decision tree was derived from the PGM, employing a custom-built algorithm designed to translate the PGM’s probabilistic reasoning into a transparent rule-based classifier. The algorithm iteratively evaluated combinations of the five key input variables, seeking optimal division of the high-risk PCa patients into three distinct risk subgroups for PD—low- and intermediate risk vs. highest risk—guided by a minimum probability separation of 0.2 for clarity and clinical relevance (see Figure 6). For a detailed description of the decision tree development process, see Appendix A.

## 3. Results

### 3.1. Input Variable Assessment

**Figure 3:**
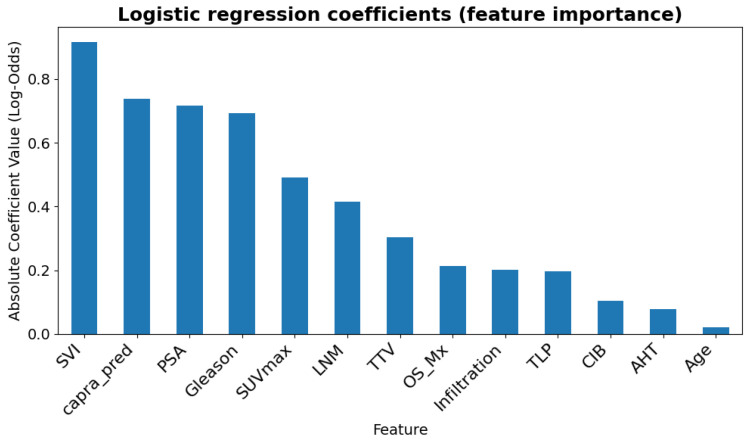
Logistic regression coefficients are presented in absolute values. The SVI, CAPRA, PSA, Gleason score, and SUVmax showed the strongest relationship with PCa progression.

The four input variables with the highest relationship to PCa progression were the SVI, CAPRA, PSA, and the Gleason score, with standardized absolute coefficients of 0.92, 0.74, 0.71, and 0.70, respectively. The SUVmax, LNM, TTV, and OS_Mx, showed lower absolute coefficients of 0.49, 0.41, 0.30, and 0.21, respectively, indicating their relevance despite weaker effects. The remaining features exhibited minimal predictive contibution, with standardized absolute value coefficients <0.2 (see Figure 3). However, it is important to note that the PSA, SVI, Gleason, and LNM were already included in the capra_pred calculations.

**Figure 4:**
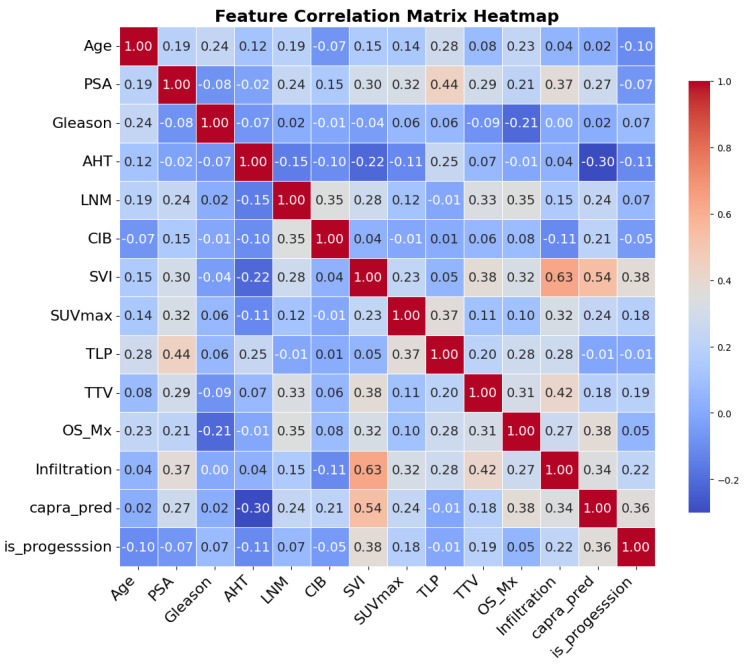
Correlation matrix of input variables. The analysis confirmed low inter-feature correlation overall, supporting their joint inclusion in the model, with only SVI and extracapsular infiltration showing moderate overlap (r = 0.64).

The correlation matrix, visualized in Figure 4, revealed a moderate to strong positive correlation between SVI and extracapsular infiltration (r = 0.64), indicating redundancy between these two potential input variables. In contrast, most other feature pairs showed low to moderate correlations (|r| < 0.6), suggesting acceptable independence for inclusion in our models.

Spearman rank correlation analysis revealed statistically significant positive correlations between early PCa progression and both SVI (ro = 0.381, *p* < 0.001) and the predicted CAPRA score (ro = 0.363, *p* < 0.001). None of the other input variables tested showed a significant correlation with early PCa progression (all *p* > 0.05).

### 3.2. Input Variable Selection

A set of five key input variables was incorporated into our predictive models. Each variable was encoded as binary and assigned a weight reflecting its relative importance (0.5–1.5) in the PGM as described in Table 5. The features age, PSA, LNM, and Gleason score were not modeled independently, as they are subsumed within the CAPRA score. The TTV was not incorporated into the predictive models to avoid the complexities of discretization despite its significance in the ranking (standardized absolute coefficient 0.30).

The final weight assignments—ranging from 0.5 to 1.5—were based on domain knowledge and the existing literature, not arbitrary choices. The binarized CAPRA score was assigned the highest weight (1.5) to reflect its validated prognostic utility for multiple outcomes, including eBCR and metastasis [27]. SUVmax was weighted at 1.0 based on its recognized context-dependent value as a non-invasive biomarker of tumor aggressiveness and progression risk [28,29]. A further rationale for those particular features is outlined in the discussion and Appendix A.

### 3.3. Model Comparison

The LR model yielded a specificity of 1.0, but it did not identify any true positive cases, resulting in zero sensitivity and a balanced accuracy of 0.5.

In contrast, the PGM showed substantially higher discriminative ability. The model achieved a sensitivity of 0.6 and a specificity of 0.864, resulting in a balanced accuracy of 0.732, the highest among all models tested.

The CAPRA-based score as a standalone comparator yielded only a low sensitivity of 0.2; however, it had the highest specificity of 0.989, resulting in a balanced accuracy of 0.594.

**Figure 5:**
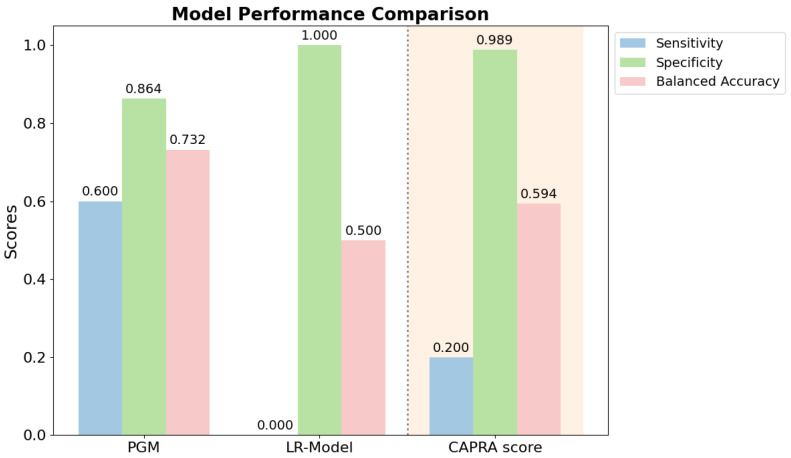
Comparative performance of the LR model and PGM with the CAPRA score as standalone comparator with the respective key validation metrics. The PGM shows the best balance between sensitivity and specificity, while the LR model and the CAPRA score demonstrate perfect specificity at the cost of sensitivity.

A comparison of all models based on discriminative performance metrics is provided in Figure 5.

### 3.4. Decision Tree Development

**Figure 6:**
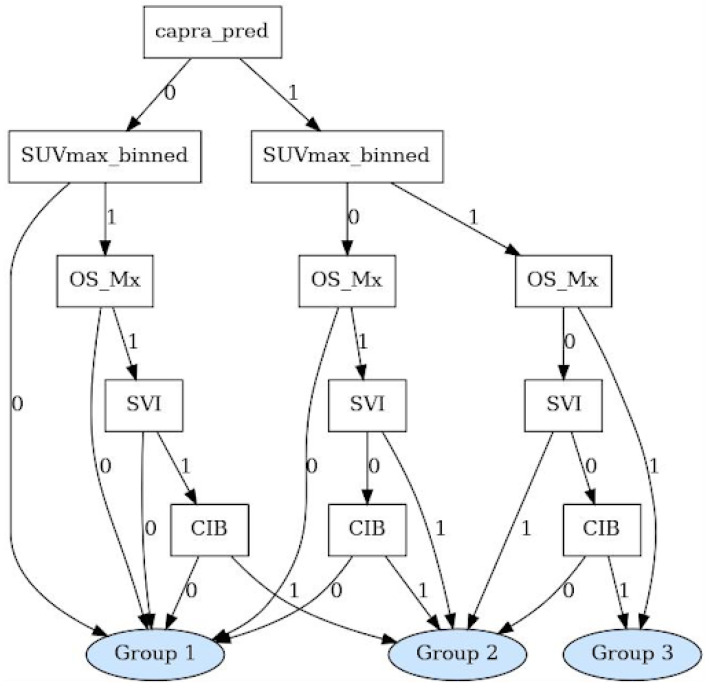
The decision tree uses binary input variables, where a value of 0 indicates a negative or low-risk finding. Specifically, SUVmax_binned = 0 denotes a primary tumor SUVmax below 12; capra_pred = 0 indicates a CAPRA-based progression probability below 0.15. For the remaining variables, a value of 0 corresponds to the absence of the respective pathological feature: no bone metastases (OS_Mx), no seminal vesicle infiltration (SVI), and no lymph node involvement at the common iliac bifurcation (CIB).

The decision tree structure that stratifies high-risk patients in three distinct progression risk groups is shown in Figure 6. A CAPRA score > 0.15 was identified as the first and most influential split, followed by SUVmax ≥ 12 as the second decision node. Patients with high CAPRA scores, elevated SUVmax, and bone metastasis or LNM at CIB were consistently classified in the highest-risk group for early PCa progression. Additional stratification was provided by the presence of bone metastases, CIB nodal involvement, and SVI as a separate node, with combinations of these characteristics defining the assignment of the risk group. The resulting classification assigned 7.5% of the patients to the highest risk group (Group 3), 19.3% to the intermediate risk (Group 2), and 73.1% to the lowest risk group (Group 1) in our cohort of high-risk patients. The risk distribution aligns with established prognostic markers and supports its potential clinical utility. In particular, the decision rules used to define each group mirrored the existing clinical intuition.

## 4. Discussion

Clinicians require reliable tools to identify patients at high risk of early PCa recurrence after primary treatment. Commonly used clinical risk stratification systems, such as the D’Amico, NCCN, AUA, or CAPRA scores, effectively leverage clinical data and classify patients into broad risk groups. However, their trade-off is large intragroup heterogeneity and reduced prognostic precision due to the dichotomization of continuous variables [30,31]. In addition, these systems do not incorporate advanced molecular imaging findings, such as ^68^Ga-PSMA-11, which have demonstrated superior diagnostic and prognostic capabilities [10,11,12]. This creates an opportunity for predictive models using ML to leverage more granular multimodal data and overcome the limitations of traditional clinical risk tools, as we showed in our study.

### 4.1. Model Comparison

The PGM, trained with expectation maximization, demonstrated the highest discriminative ability compared to the LR model and the CAPRA score. It shows the potential of data- and expert-driven ML approaches to predict early PCa progression, although such cases are relatively rare, and underscores the importance of keeping these technologies at the forefront of clinical focus. The inability of the LR model to detect true positive cases likely reflects the limitation of the model in handling the class imbalance present in our dataset, where progression events were <10%. For comparison, a rule-based approach using a clinical risk score was also assessed. Although the CAPRA score achieved high specificity, its limited sensitivity resulted in only moderate overall performance. These findings suggest that although CAPRA lacks sufficient sensitivity to detect early progression, it remains valuable as a baseline risk stratification tool.

However, despite the superior performance of PGM, its relevance and acceptance in clinical routine practice should be evaluated in larger studies, particularly its added value for experienced physicians who might rather rely on their clinical knowledge and intuition. Therefore, the performance metrics achieved in our study must be interpreted with caution and may not directly translate into clinical decision support without consistent case-level applicability. Prospective validation of the PGM in a larger patient cohort that focuses on interpretability and real-world performance is essential.

### 4.2. The Impact of Molecular Imaging for Predictive Models

Molecular diagnostics are poised to play a pivotal role not only in guiding therapy decisions but also in shaping modern risk stratification strategies. In a regression-based study by Djaileb et al. [15], a high SUVmax was associated with shorter BCR-free survival in univariate analysis, but it lost importance in multivariate models that included CAPRA scores and PSMA-detected extraprostatic disease. The authors concluded that SUVmax within the prostate did not independently improve risk stratification beyond established clinical tools. In contrast, our ML-based approach found SUVmax to be a key predictor in the PGM, second only to the CAPRA score, and integrated it into a decision tree. Applying a binary threshold to SUVmax (≥12), the PGM yielded high sensitivity, specificity, and balanced accuracy. We conclude that SUVmax adds a significant predictive value for early progression of PCa, even if anatomical and clinical characteristics are considered. The discrepancy between our results regarding the importance of SUVmax and the findings of Djaileb et al. could be attributed to differences in modeling approaches, as well as the sample size, duration of follow-up, and patient risk profiles.

Other results of our study are consistent with previous findings, showing that the superior diagnostic performance of PSMA-PET imaging improves prognostic precision for PCa recurrence, particularly by demonstrating extraprostatic tumor spread. Djaileb et al. [15] identified extraprostatic disease (N1/M1) as the strongest independent predictor of recurrence. The superior performance of ^68^Ga-PSMA-11 PET/CT for the localization of extraprostatic tumor growth and distant spread directly contributed to the performance of our models. Inclusion of SVI, lymph node, and bone metastases as weighted input variables improved the specificity of the PGM. The SVI has long been associated with a poor prognosis and additional lymph node involvement [32]. By treating SVI as an independent predictor—rather than embedding it within a composite score such as CAPRA-S—our model emphasizes the evidence that SVI is strongly associated with poor therapeutic outcomes [33]. Evidence links extrapelvic (retroperitoneal) nodal metastases detected by PSMA-PET to a substantially higher risk of early escalation of treatment—up to five times within 29 months [34]. However, our results suggest that the critical anatomical threshold for identifying patients with very high risk for early PCa progression may lie more proximally to the prostate, at the CIB level, rather than in the retroperitoneal region. The inclusion of nodal involvement at the CIB as a separate input variable further highlights the importance of detailed anatomical information for recurrence prediction derived by ^68^Ga-PSMA-11 PET/CT. It is also an essential factor for effective planning of local therapy [35].

### 4.3. Comparison of PGM to Other PSMA-PET Risk Calculators

A strength of our PGM lies in its integration of established and widely used clinical risk scores such as CAPRA along with quantitative PSMA-derived imaging features such as SUVmax and anatomical data. Thus, it extends other models such as the MRI-PSMA risk calculator [36] or the PSMA-PROMISE nomogram [37], which use primarily quantitative measurements such as the SUVmean and anatomical data such as lymph node, bone, and organ metastases. The combination of established clinical risk scores and imaging biomarkers for the prediction of tumor recurrence represents a logical progression toward a more comprehensive risk stratification [38,39].

A key aspect of this evolution is the further integration of PSMA-derived radiomics into a multi-omics framework—an approach that combines imaging-derived features with biomolecular data, such as genomic, proteomic, or metabolomic profiles, to enable more holistic models for risk stratification by linking imaging phenotypes to their underlying molecular characteristics [40,41].

### 4.4. Limitations

Several relevant limitations of our approach merit attention. The retrospective nature and the moderate sample size of the dataset (n = 93) may introduce systematic error and reduce the reproducibility of the study.

A potential limitation could be the use of [68Ga]Ga-PSMA-11 due to its high bladder uptake, which might limit the detectability of findings at the base of the prostate or in the seminal vesicles. Other [18F]F-labeled PSMA ligands, such as [18F]F-PSMA-1007, can offer advantages in delineating local recurrence and pelvic lymph node metastases due to their hepatobiliary excretion, which reduces urinary interference [42]. However, [68Ga]Ga-PSMA-11 is used as a standard of care diagnostic agent in our institution due to its rapid pharmacokinetics, demonstrating low retention in non-tumor tissues, which improves image clarity and diagnostic accuracy [43]. The disadvantage of higher bladder uptake seen with [68Ga]Ga-PSMA-11 can be minimized by retrospective anatomical correlation with diagnostic CT scans or, ideally, with multiparametric MRI, which was available in most cases at the time of initial staging.

In cases like ours, purely data-driven feature selection methods present significant challenges [44,45]. In such a small sample, automated techniques may struggle to reliably identify truly discriminative variables for the minority class, potentially leading to unstable or biased models [44]. Therefore, integrating clinical experience and literature review into the feature selection process was crucial to ensure that the selected variables were clinically relevant and robust despite data limitations [45].

Furthermore, the short and variable follow-up period, in some cases limited to only 30 days after local therapy, can affect progression classification. The decision to include these patients was clinically motivated, as PSA levels are generally expected to become undetectable within four weeks after complete resection, and persistent PSA can indicate residual disease or early recurrence [46]. Thus, keeping in line with the current consensus of the European PCa Guidelines Panel—2024 Update [3], it seems plausible to include patients with short follow-up.

Based on previous findings that combining presurgical CAPRA scores with PET-derived features from PSMA produces predictive performance comparable to postsurgical CAPRA-S [15], we extended this approach by using PSMA-based findings to impute missing components of CAPRA-S, advancing the integration of imaging-based surrogates into this established clinical risk model. The use of [^68^Ga]Ga-PSMA-11 to infer pathological variables such as SVI and nodal status is supported by the literature, given the high specificity of PET for these characteristics relative to histopathology [47]. However, our approach, although advocated by other authors, has yet to be sufficiently validated, since other studies have shown only moderate concordance between imaging-based evaluations and pathological reference standards [48].

More recently, minimally invasive liquid biopsies, which are complementary to advanced functional imaging, have been integrated into the NCCN guidelines, adding prognostic value to traditional tissue sampling and clinical evaluation [49,50]. However, liquid biopsies are not yet integrated into the German S3 PCa Guideline and thus are not considered standard clinical care. Since our study comprised retrospective data, liquid biopsy results were not available.

## 5. Conclusions and Further Directions

Our study demonstrates that integrating imaging biomarkers derived from ^68^Ga-PSMA-11 PET/CT, such as intraprostatic SUVmax and established clinical risk scores, significantly improves the prediction of early progression of PCa after primary therapy. Compared to traditional statistical methods such as LR models, our ML-based PGM and its derived decision tree achieved superior balanced accuracy—even within a moderately sized and unbalanced cohort of patients with high-risk PCa.

Prospective validation focusing on interpretability and real-world performance of the PGM in a larger cohort of patients remains essential. To ensure the generalizability of our results, future efforts should also prioritize multiinstitutional data collection, standardized imaging protocols, and external validation in prospective randomized trials.

## Figures and Tables

**Table 1:** Baseline patient characteristics. Patients exhibited a broad range of PSA values at diagnosis, reflecting the heterogeneity typical for high-risk PCa. Similarly, [^68^Ga]Ga-PSMA-11 imaging revealed high variability in intraprostatic SUVmax values, indicating substantial differences in tumor PSMA expression within this patient group.

Characteristic	Median	Range
Age (years)	67.0	52–83
PSA (ng/mL)	10.5	0.01–153
Gleason score	8	6–10
SUVmax	21.44	4.18–154.95
TLP	29.35	1.25–3651.1
TTV (mL)	5.13	0.35–115.15

**Table 2:** [^68^Ga]Ga-PSMA-11 imaging findings. Bone metastases (OS_Mx) were detected in 12.8% of patients, while any lymph node metastases (LNM) were present in 26.9% of cases. Seminal vesicle infiltration (SVI) was observed in 18.3% of patients.

Variable	Positive	Negative
Bone metastases (OS_Mx)	12 (12.8%)	81 (87.1%)
LNM at common iliac bifurcation	4 (4.3%)	89 (95.7%)
LNM at internal iliac artery (IIA)	11 (11.8%)	82 (88.2%)
LNM at external iliac artery (EIA)	19 (20.4%)	74 (79.6%)
LNM at common iliac artery (CIA)	10 (10.7%)	83 (89.2%)
Seminal vesicle infiltration (SVI)	17 (18.3%)	76 (81.7%)
LNM retroperitoneal	20 (21.5%)	59 (63.4%)

**Table 3:** Treatment outcomes. The outcomes were categorized into response groups. The majority of patients achieved either complete response (CR, 59.1%) or partial response (PR, 16.2%), while 19.4% had stable disease (SD) and 5.4% showed progressive disease (PD). This distribution reflects a generally favorable treatment response profile within the high-risk patient cohort.

Clinical Outcome	Number of Patients (%)
Complete response	55 (59.1%)
Stable disease	18 (19.4%)
Partial response	15 (16.2%)
Progression	5 (5.4%)

**Table 4:** Potential predictive input variables considered for model development.

Category	Variable	Description
Clinical	Age	patient age at diagnosis
	PSA	baseline prostate-specific antigen level (ng/mL)
	Gleason score	histopathologic grade group
	CAPRA-S/J-CAPRA (capra_pred)	continuous CAPRA-derived risk score
	Primary treatment modality	treatment type at baseline (e.g., surgery, adjuvant hormone therapy (AHT))
PET Imaging Features	SUVmax	maximum standardized uptake value
	Total lesion PSMA uptake (TLP)	summed uptake volume across all lesions
	Total tumor volume (TTV)	volumetric measure of the main tumor
	Bone metastases (OS_Mx)	presence of PSMA-positive skeletal lesions
	Lymph node metastasis (LNM)	nodal uptake on PSMA PET across anatomical regions
	– Internal iliac artery (IIA)	
	– External iliac artery (EIA)	
	– Common iliac bifurcation (CIB)	
	– Common iliac artery (CIA)	
	– Retroperitoneal	
	Seminal vesicle infiltration (SVI)	tracer uptake visible in this anatomical region
	Periprostatic infiltration	extension of tracer uptake beyond prostate capsule

**Table 5:** Input variables and assigned weights. The same five binary variables were used in the LR model and PGM. In the PGM, weights were assigned to the input variables based on evidence and derived through iterative performance optimization. CAPRA and SUVmax received the highest weights. SVI, OS_Mx, and CIB represented key anatomical and metastatic features.

Feature (LR, PGM)	Description	Weight (PGM)
CAPRA score (capra_pred)	Binarized recurrence risk derived from survival curves (threshold = 0.16)	1.5
SUVmax	Binarized intraprostatic PSMA uptake, threshold at 12.0 (SUVmax_binned)	1.0
Seminal vesicle infiltration (SVI)	Binarized (yes/no), histopathologically proven or inferred via PSMA-imaging	0.5
Bone metastases (OS_Mx)	Binarized (yes/no), presence of PSMA-positive bone lesions	0.5
Common iliac node involvement (CIB)	Binarized (yes/no), presence of PSMA-avid lymph nodes at common iliac bifurcation	0.5

## Data Availability

Data can be shared on request if appropriate documents will be signed between institutions of interest.

## Appendix A

### Appendix A.1. Feature Engineering

For feature engineering to address missing data and integrate prior to model development, preprocessing of the dataset comprised the removal of duplicate entries, the exclusion of incomplete datasets, the verification of key variables, and the manual correction of extreme outliers if necessary.

Due to missing data in certain fields, structured feature engineering steps were applied. For CAPRA-S, the surgical margin status was not available and was approximated by averaging the score assuming both positive and negative margins. Information missing from stage T was inferred from [^68^Ga]Ga-PSMA-11: seminal vesicle infiltration was used to approximate the T3b status, while infiltration of the rectum or retroperitoneal space was considered indicative of T4. Additional missing variables—seminal vesicle involvement, extracapsular extension, and nodal status—were also imputed using PET/CT findings when absent from clinical histopathological records. PSA was grouped into discrete intervals (0–6, 6.01–10, 10.01–20, >20 ng/mL), and Gleason grade groups were assigned based on clinical pathology (2–6, 3 + 4, 4 + 3, 8–10).

J-CAPRA scores were generated similarly using binned PSA values (0–20, 20–100, 100–500, >500), Gleason score groupings (2–6, 7, 8–10), clinical T stage, and nodal/metastatic staging, with missing entries inferred from PET-based anatomical information.

Since both CAPRA systems typically report long-term risk in discrete time intervals (e.g., 5 or 10 years), per-patient progression probabilities were obtained by extracting data from published survival curves: CAPRA-S from Cooperberg et al. and J-CAPRA from Shiota et al. These probabilities were then binarized using a 0.16 threshold, empirically determined by iterative evaluation of sensitivity, specificity, and balanced accuracy. This binary output served as the primary clinical predictor in the model. Capra_score was set to equal the j_capra_score column if the value of AHT column is 1 and to capra_s_score otherwise.

### Appendix A.2. Integration of Imaging-Derived Biomarkers and Input Variables Selection

The selection of the input variables for the models was based on a combination of statistical analysis, domain expertise, and the current literature. In the end, five binary variables were incorporated into the final predictive models: binarized CAPRA score, SUVmax (≥12), presence of bone metastases (OS_Mx), lymph node involvement at the common iliac bifurcation (CIB), and seminal vesicle infiltration (SVI). These variables were chosen based on their prognostic relevance, low intercorrelation, and interpretability in a clinical context.

CAPRA score (capra_pred), binarized at a progression probability threshold of 0.16, served as the primary clinical predictor. It integrated age, PSA, Gleason score, T stage, and nodal involvement and was recalculated for all patients using standardized scoring rules. The missing components of CAPRA, including surgical margin status and clinical staging variables, were imputed using the [^68^Ga]Ga-PSMA-11 findings. For example, SVI and rectal involvement on PET/CT were used to approximate T3b and T4 disease, respectively, and dual-assumption imputation was applied for surgical margins, mirroring contemporary multiple imputation strategies [47].

SUVmax was selected as a key imaging biomarker due to its high reproducibility and strong evidence base in PCa studies. Although there is no universal threshold, reports in the literature have proposed SUVmax cutoffs ranging from 5.6 to 9.1 to predict high-risk disease or recurrence [51,52,53]. Our model used a threshold of 12, reflecting both the high-risk characteristics of our cohort and the previous findings that this value achieves high specificity for clinically significant PCa [51]. SUVmax was assigned a relative weight of 1.0, second only to CAPRA (1.5), and emerged as the second most influential variable in the decision tree.

SVI was modeled as an independent variable despite its partial overlap with the CAPRA scoring system. This decision was supported by its high coefficient in logistic regression, consistent evidence of prognostic value in surgical cohorts [32,33,54], and improved model performance in early validation folds. Including SVI as a distinct feature aligns with the literature that shows its strong association with biochemical recurrence and poor survival.

Bone metastases were included as a binary input variable with a weight of 0.5, supported by strong evidence linking skeletal involvement to reduced overall survival and aggressive disease behavior [14,55,56]. PET-based quantification of PSMA bone lesions has shown risk ratios that exceed 3.9, strengthening its inclusion as a prognostic determinant in our study [56]. The involvement of lymph nodes in the common iliac bifurcation (CIB) was selected to enhance anatomical granularity. This site has gained attention in the recent literature for its potential prognostic importance and role in early treatment escalation decisions [34,35]. CIB status was modeled as a discrete feature with a weight of 0.5, aligning with clinical utility to guide surgery treatment and radiotherapy field planning.

The variable “days_diff” captures the time interval in days between baseline [^68^Ga]Ga-PSMA-11 and clinical outcome assessment, as recorded in the variable “overall_last_status”. We present the complete distribution of patient responses in Table 3 to maintain transparency with respect to the original data structure and to inform future studies that may pursue more granular outcome predictions in larger cohorts.
